# Effect of Oral and parenteral routes of vitamin D supplementation on serum 25(OH) vitamin D levels in patients with non-alcoholic fatty liver disease

**DOI:** 10.22088/cjim.13.1.23

**Published:** 2022

**Authors:** Arezou Hamzehzadeh Alamdari, Samira Ahrabi, Manouchehr Khoshbaten, Shahram Roustaei, Sara Araqchin Ahrabi, Mohammad Asghari Jafarabadi

**Affiliations:** 1 Gastroenterology and Liver Diseases Research Center, Department of Internal Medicine, Tabriz University of Medical Sciences, Tabriz, Iran; 2 Department of Surgery, Tehran University of Medical Sciences, Tehran, Iran; 3 Department of Statistics and Epidemiology, School of Medicine, Zanjan University of Medical Sciences, Zanjan, Iran; 4 Center for the development of interdisciplinary research in Islamic sciences and health sciences, Tabriz University of Medical Sciences, Tabriz, Iran

**Keywords:** Nonalcoholic fatty liver disease, Vitamin D, Bile acid, Routes of administration

## Abstract

**Background:**

Due to the interruption of the EHC pathway in NAFLD patients, we hypothesized that parenteral vitamin D supplementation is superior to oral in vitamin D insufficient patients with NAFLD. Therefore, this study aimed to compare the efficacy of oral and parenteral routes of vitamin D supplementation on serum 25(OH) vitamin D levels in patients with NAFLD.

**Methods:**

In this prospective randomized trial, 66 NAFLD cases with vitamin D deficiency were studied. For 33 cases, oral vitamin D was supplemented, whereas the other 33 patients were given an intramuscular injection of vitamin D. Laboratory tests *and liver ultrasound were performed at the beginning and the end of the trial for each subject.*

**Results:**

Regardless of the drug administration route, at the end of this trial the mean of serum 25-hydroxy *vitamin D level increased from* 8.74±2.47 to 33.16±17.61 (P=0.00), and the mean±SD for serum triglyceride decreased from 191.46±92.79 to 166.00±68.30 (P=0.02), both were statistically significant. *Liver ultrasound reported statistically significant changes in the grade of fatty liver disease* *(P=0.003).* In the comparison between the two groups, serum 25-hydroxy *vitamin D level changes were not statistically significant (P=0.788).*

**Conclusion:**

The intramuscular method of supplementation was not better than the oral route in improving serum 25(OH) vitamin D levels in NAFLD patients. In this study, the impaired EHC and vitamin D absorption inhibitor factors in NAFLD patients did not affect the final result of serum vitamin D levels significantly.

Non-alcoholic fatty liver disease (NAFLD) is characterized by the accumulation of excessive fat in hepatocytes and is considered as the hepatic component of the metabolic syndrome, because of the insulin resistance that they have in common in the pathophysiology (1, 2). It represents the spectrum of liver problems in different four stages: simple fatty liver (Steatosis), nonalcoholic steatohepatitis (NASH), fibrosis, and eventually cirrhosis at the most severe stage (3, 4). NAFLD is a highly prevalent condition affecting 25% of the general adult population and even two times more common in the population affected by metabolic syndrome (5). It is becoming a major health issue as the evidence has proven that beyond mortality and morbidity associated with liver-related diseases such as cirrhosis, liver failure, and hepatocellular carcinoma, it also adversely influences several body systems and can lead to kidney diseases, cardiovascular diseases, and type 2 diabetes mellitus (2, 6). Interestingly, recent studies have indicated that alteration in the composition of bile acids (BAs), as well as serum BAs level were closely associated with NAFLD development (7-11). 

BAs are amphipathic steroid molecules derived from cholesterol by the liver. Besides assisting micelle formation and facilitating the emulsification, absorption, and transportation of lipids and lipophilic vitamins (12), BAs act as hormone-like signaling molecules and regulate metabolic pathways of cholesterol, lipids, and glucose through modulating bile acid nuclear receptors such as Farnesoid X receptor (FXR) and Takeda G-protein-coupled receptor 5 (TGR5) (9, 13-15). 

Learning the action of these receptors suggests the role of BAs in NAFLD pathogenesis. In a recent study that measured serum bile acid composition in NAFLD patients, an increase in FXR antagonists and a decrease in FXR agonists levels were reported (16). These altered bile acid compositions are pertinent to metabolic homeostasis. As a result, evidence indicates the association between blood BAs and NAFLD and shows the role of disturbances within the enterohepatic circulation (EHC) pathway in the development of liver dysfunction (9, 13, 16, 17).

On the other hand, vitamin D deficiency, which afflicts about 73% of the world and 42% of the USA population (18, 19), is often accompanied by NAFLD and plays an important role in its pathogenesis and progression. Many studies have indicated a strong association between vitamin D deficiency and NAFLD. The accurate pathogenesis of this association is still unclear (20, 21). 

However, evidence from animal studies points the finger at an impaired EHC, through which vitamin D deficiency can upregulate endogenous fatty acid synthesis into NASH and suggests that 1,25(OH)_2_VD_3_ (calcitriol) administration may correct the NASH (22). Although all the guidelines of management of NAFLD have emphasized the remarkable effects of lifestyle on the improvement of patients' condition, current guidelines point toward vitamin D as a potential therapeutic option for these patients (23-27). 

The importance of improvement of serum vitamin D status in NAFLD cases and on the other hand interruption of the EHC pathway in these patients, which can affect the absorption of oral vitamin D, encouraged us to conduct this research. Due to the interruption of the EHC pathway in these patients, we hypothesized that parenteral vitamin D supplementation is superior to oral in vitamin D insufficient patients with NAFLD. Therefore, this study aimed to compare the efficacy of oral and intramuscular routes of vitamin D supplementation on serum 25(OH) vitamin D levels in patients with NAFLD.

## Methods

This study was approved by the Ethics Committee of Tabriz University of Medical Sciences (Code: IR.TBZMED.REC.1398.882), and written informed consent was obtained from all the participants. This prospective, randomized, single-institution clinical trial, in which 66 NAFLD cases with vitamin D deficiency were studied and was carried out from February to Jun 2020, in the Liver Research Center, Tabriz, Iran. 

Eligible participants were NAFLD cases, confirmed with abdominal ultrasound, older than 18-year-old, and had serum 25-hydroxyvitamin D levels less than 20 ng/ml. First, participants on calcium or vitamin D supplementation in the past 2 months, subjects on any medication which can cause liver damage or liver enzymes elevation, and patients with cirrhosis, hepatitis, thyroid or parathyroid disorders, and any other health conditions that can lead to secondary vitamin D deficiency were excluded. 

Then, blood samples were collected from each patient and serum 25-hydroxy vitamin D level, lipid profile, liver enzymes, and fasting blood sugar level were measured. Liver ultrasonography was performed to specify the initial grade of NAFLD. 

Next, subjects were randomized into one of the following two groups: Group 1, included 33 cases (13 men and 20 women, age mean±standard deviation (SD), 51.30±10.78), received oral cholecalciferol 50,000 IU weekly for four weeks followed by 50,000 IU monthly for two months. The other half of the subjects, group 2 (20 men and 13 women, age mean±SD, 48.67±10.31), were given an intramuscular injection of cholecalciferol 300,000 IU, monthly for three months. 

During the trial, all the participants were asked to maintain their usual lifestyle and habitual dietary intake. Four months after the beginning of the trial, laboratory tests and liver ultrasounds were repeated in all subjects.

Finally, the statistical analysis of this study was conducted using IBM SPSS statistics software Version 26. All categorical variables were reported as frequency and percentage, and numerical variables were reported as mean ± SD. Chi-square test and t-test were used to analyze categorical and quantitative variables, respectively. Comparing data before and after vitamin D administration was performed by Paired t-test for the quantitative data and Wilcoxon test for the categorical data. P-values less than 0.05 were considered statistically significant.

## Results

Overall, 66 (33 men and 33 women; age range 22-69 and mean±SD, 49.98±10.55) cases were enrolled in this study. Among the included cases, 33 (13 men and 20 women, age mean±SD 51.30±10.78) cases were in group 1 and 33 (20 men and 13 women, age mean±SD, 48.67±10.31) cases were in group 2. As noticeable in table 1, which provides demographic, laboratory, and liver ultrasound findings at the beginning of the trial, there were no significant differences between groups regarding age, sex, baseline liver ultrasound, and laboratory findings. Table 2 reflects the data at the end of the study. The initial mean ± SD for serum level of 25-hydroxy vitamin D for all subjects was recorded as 8.74±2.47. Baseline ultrasound reports revealed grade one for 64%, grade two for 35%, and grade three fatty liver disease for 1% of the participants. 

**Table 1:** Initial demographic, laboratory, and liver ultrasound findings

	**All patients** **(N=66)**	**Group 1** **(N=33)**	**Group 2** **(N= 33)**	**P**
Age (Y)	49.98 ±10.55	51.30 ± 10.78	48.67 ± 10.31	0.31
Sex				0.07
Female Male	33 (50%) 33 (50%)	20 (61%) 13 (39%)	13 (39%) 20 (61%)	
Grading				0.14
Grade 1 Grade 2 Grade 3	42 (64%) 23 (35%) 1 (1%)	18 (54.5%) 15 (45.5%) 0 (0.0%)	24 (72.7%) 8 (24.2%) 1 (3.0%)	
AST (Aspartate Aminotransferase), IU/l	24.84 ± 13.07	23.27 ± 8.54	26.42 ± 16.40	0.33
ALT (Alanine Aminotransferase), IU/l	31.51 ± 17.35	32.12 ± 15.47	30.90 ± 19.28	0.77
Triglycerides, mg/dl	191.46 ± 92.79	189.03 ± 104.38	193.90 ± 81.14	0.83
Cholesterol, mg/dl	185.36 ± 40.00	179.48 ± 44.19	191.24 ± 35.02	0.23
Fasting Blood sugar, mg/dl	97.18 ± 24.16	99.40 ± 28.29	94.96 ± 19.37	0.46
25-hydroxyvitamin D, ng/dl	8.74 ± 2.47	8.19 ± 2.60	9.29 ± 2.25	0.06

Note: Except for sex and grading (number of patients with the percentage in parenthesis) other data is mean ± standard deviation.

**Table 2:** Final laboratory and liver ultrasound findings

	**All patients** **(N=66)**	**Group 1** **(N=33)**	**Group 2** **(N= 33)**	**P**
Grading Normal Liver Ultrasound Grade 1 Grade 2 Grade 3	12 (18%) 36 (55%) 17 (26%) 1 (1 %)	7 (21%) 15 (45%) 10 (30%) 1 (3%)	5 (15%) 21 (64%) 7 (21%) 0 (0%)	0.00
AST (Aspartate Aminotransferase), IU/l	24.74 ± 11.62	24.42 ± 12.58	25.06 ± 10.76	0.55
ALT (Alanine Aminotransferase), IU/l	19.04 ± 16.23	29.09 ± 18.82	29.00 ± 13.46	0.52
Triglycerides, mg/dl	166.00 ± 68.30	163.63 ± 64.22	168.36±73.08	0.11
Cholesterol, mg/dl	187.80 ± 36.96	191.75 ± 37.33	183.84±36.73	0.24
Fasting Blood sugar, mg/dl	102.46 ± 30.39	104.75 ± 38.95	100.18±18.64	0.08
25-hydroxyvitamin D, ng/dl	33.16 ± 17.61	33.09 ± 20.26	33.24±14.83	0.00

Note: Except for fatty liver disease grading (number of patients with the percentage in parenthesis) other data is mean ± standard deviation.

Regardless of the drug administration route, at the end of this trial, the mean of serum 25-hydroxy vitamin D level increased from 8.74±2.47 to 33.16±17.61 (P=0.00) and the mean±SD for serum triglyceride decreased from 191.46±92.79 to 166.00±68.30 (P=0.02), both were statistically significant. Liver ultrasound reported no change in the grade of NAFLD in 40 cases. However, 6 cases were reported changing to the upper grade and 20 cases to the lower grade which were statistically significant (P=0.003).

Regarding drug administration route, in group 1 subjects, who were under oral cholecalciferol supplementation, the mean of serum 25-hydroxy vitamin D level increased from 8.19±2.60 to 33.09±20.26 (P=0.00), and in group 2 patients, who underwent intramuscular injection, it was raised from 9.29±2.25 to 33.24±14.83 (P=0.00), which was statistically significant in either group. 

The other laboratory data did not change significantly in any of the groups (tables 3 and 4). In the comparison between the two groups, serum 25-hydroxy vitamin D level changes were not statistically significant (P=0.788).

**Figure 1. A:**
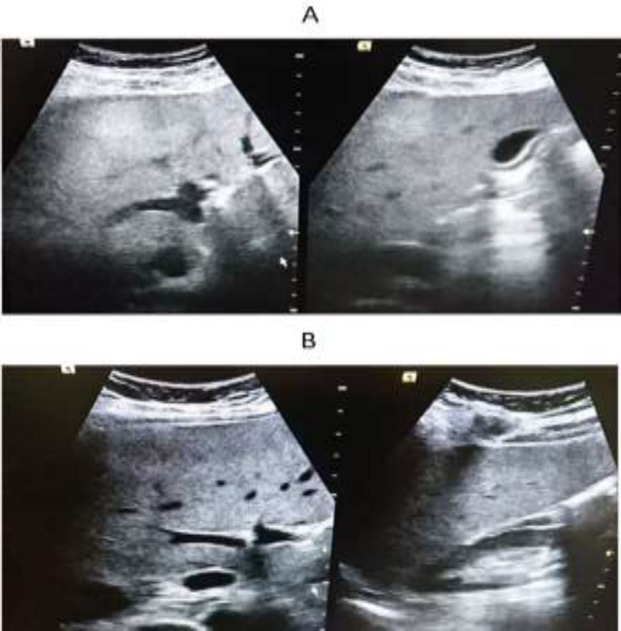
The initial liver ultrasound in a 56-year-old man shows fatty liver disease grade three. B: Repeated ultrasound after three months of vitamin D administration shows fatty liver disease grade one

**Table 3:** Comparison of initial and final laboratory and sonographic data in patients who received oral cholecalciferol

	**Initial Data**	**Final Data**	**P**
Grading			0.25
Normal Liver Ultrasound Grade 1 Grade 2 Grade 3	0 (0.0%) 18 (54.5%) 15 (45.5%) 0 (0.0%)	7 (21%) 15 (45%) 10 (30%) 1 (3%)	
AST (Aspartate Aminotransferase), IU/l	23.27 ± 8.54	24.42 ± 12.58	0.53
ALT (Alanine Aminotransferase), IU/l	32.12 ± 15.47	29.09 ± 18.82	0.25
Triglycerides, mg/dl	189.03 ± 104.38	163.63 ± 64.22	0.11
Cholesterol, mg/dl	179.48 ± 44.19	191.75 ± 37.33	0.18
Fasting Blood Sugar, mg/dl	99.40 ± 28.29	104.75 ± 38.95	0.34
25-hydroxyvitamin D, ng/dl	8.19 ± 2.60	33.09 ± 20.26	0.00

Note: Except for fatty liver disease grading (number of patients with the percentage in parenthesis) the other data is mean ± standard deviation.

**Table 4:** Comparison of initial and final laboratory and sonographic data in patients who received intramuscular cholecalciferol

	Initial Data	Final Data	P
Grading			0.59
Normal Liver Ultrasound Grade 1 Grade 2 Grade 3	0 (0%) 24 (72.7%) 8 (24.2%) 1 (3.0%)	5 (15%) 21 (64%) 7 (21%) 0 (0%)	
AST (Aspartate Aminotransferase), IU/l	26.42 ± 16.40	25.06 ± 10.76	0.55
ALT (Alanine Aminotransferase), IU/l	30.90 ± 19.28	29.00 ± 13.46	0.52
Triglycerides, mg/dl	193.90 ± 81.14	168.36 ± 73.08	0.11
Cholesterol, mg/dl	191.24 ± 35.02	183.84 ± 36.73	0.25
Fasting Blood Sugar, mg/dl	94.96 ± 19.37	100.18 ± 18.64	0.09
25-hydroxyvitamin D, ng/dl	9.29 ± 2.25	33.24 ± 14.83	0.00

Note: Except for fatty liver disease grading (number of patients with the percentage in parenthesis) other data is mean ± standard deviation.

## Discussion

Considering impaired BAs homeostasis and EHC in NAFLD cases, comparing the efficacy of oral and intramuscular routes of vitamin D supplementation on serum vitamin D in these patients was the main outcome of interest. Additional outcomes studied were the effects of vitamin D deficiency treatment in these subjects. To the best of our knowledge, this is the first study assessing different routes of vitamin D administration effects on serum vitamin D levels in NAFLD patients. This study suggested no significant difference in improving serum vitamin D levels between oral vitamin D supplementation and intramuscular vitamin D administration in vitamin D insufficient patients with NAFLD and the parenteral vitamin D supplementation potential superiority to oral was failed in this study. Moreover, there was no difference in the lipid profile and the grade of fatty liver disease improvement between the two groups. 

In this study, both intervention groups showed significant improvement in serum 25-hydroxy vitamin D levels at the end of the trial. However, in a comparison between the two groups, it was not statistically significant (P=0.788). The results were consistent with a similar study that was completed on healthy individuals with vitamin D deficiency and had revealed both treatment regimens significantly increased the serum 25- hydroxyl vitamin D level with a marginally significant trend in favor of oral (P=0·06) (28). The results indicate that vitamin D supplementation, regardless of the route of administration, improves serum vitamin D levels in NAFLD patients, and the impaired EHC pathway and vitamin D absorption inhibitor factors in these patients do not affect the final result significantly. 

Next, regarding the vitamin D effects on the grade of fatty liver disease, this study revealed significant improvements after three months of vitamin D supplementation (P=0.003). This can be justified by the pathogenesis that associates NAFLD and low vitamin D levels. Even though the accurate pathogenesis is still undetermined, Vitamin D level correction reduces insulin resistance in peripheral tissues and in hepatocytes, which is responsible for the NAFLD pathogenesis (21). However, the results of another study, in which 244 NAFLD patients underwent liver biopsy, revealed that vitamin D levels do not correlate with the severity of hepatic steatosis (29). In addition, no significant changes were reported for liver enzymes and glycemic index at the end of this trial, which confirmed previous studies. A systematic review of randomized controlled clinical trials, which concluded 8 articles, explained that liver enzymes did not change significantly in any of the trials on NAFLD patients after vitamin D supplementation (29).

Finally, regarding lipid profile, triglyceride levels improved at the end of this trial, which supports the mentioned systematic review results as well and could be argued as a result of the improvement of insulin sensitivity. The study had some limitations. First, we were not blinded to which interventional group each patient belonged to. Next, the follow-up period was as short as three months. Following -up with patients for a longer period of time can give us more data regarding serum vitamin D level changes throughout time. 

 In conclusion, the intramuscular method of supplementation was not better than the oral route in improving serum 25(OH) vitamin D levels in NAFLD patients. In this study, the impaired EHC and vitamin D absorption inhibitor factors in NAFLD patients did not affect the final result of serum vitamin D levels significantly.

## Funding:

This project was funded by the Tabriz University of Medical Sciences. 

## Conflict of interest:

The authors declared no potential conflict of interest with respect to the research, authorship, and publication of this article.
